# Supplementary material for: Decomposing the subclonal structure of tumors with two-way mixture models on copy number aberrations
Source: PLoS One. 2018 Dec 12;13(12):e0206579. doi: 10.1371/journal.pone.0206579 (PMC6291075; doi:10.1371/journal.pone.0206579)
Supplement: S1 Table — (PDF) [file pone.0206579.s001.pdf]

Supplementing information

S1 Table. Reference list of cell-migration-related genes

Gene Symbol	Reference
ANGPT1	[1-4]
EPHB4	[5]
FGF4	[6-8]
HAS2	[9-12]
MCC	[13, 14]
PTP4A3	[15, 16]
TGFBR2	[17-19]

1. Fagiani E, Lorentz P, Kopfstein L, Christofori G. Angiopoietin-1 and-2 exert antagonistic functions in tumor angiogenesis, yet both induce lymphangiogenesis. Cancer research. 2011;71(17):5717-27.

2. Holopainen T, Huang H, Chen C, Kim KE, Zhang L, Zhou F, et al. Angiopoietin-1 overexpression modulates vascular endothelium to facilitate tumor cell dissemination and metastasis establishment. Cancer research. 2009;69(11):4656-64.

3. Mangiola S, Hong MK, Cmero M, Kurganovs N, Ryan A, Costello AJ, et al. Comparing nodal versus bony metastatic spread using tumour phylogenies. Scientific Reports. 2016;6.

4. Li T, Yang J, Zhou Q, He Y. Molecular regulation of lymphangiogenesis in development and tumor microenvironment. Cancer Microenvironment. 2012;5(3):249-60.

5. Sharma GK, Dhillon VK, Masood R, Maceri DR. Overexpression of EphB4, EphrinB2, and epidermal growth factor receptor in papillary thyroid carcinoma: A pilot study. Head & neck. 2015;37(7):964-9.

6. Sugahara K, Michikawa Y, Ishikawa K, Shoji Y, Iwakawa M, Shibahara T, et al. Combination effects of distinct cores in 11q13 amplification region on cervical lymph node metastasis of oral squamous cell carcinoma. International journal of oncology. 2011;39(4):761.

7. Muller D, Millon R, Lidereau R, Engelmann A, Bronner G, Flesch H, et al. Frequent amplification of 11q13 DNA markers is associated with lymph node involvement in human head and neck squamous cell carcinomas. European Journal of

Cancer Part B: Oral Oncology. 1994;30(2):113-20.

8. Qi L, Song W, Li L, Cao L, Yu Y, Song C, et al. FGF4 induces epithelial-mesenchymal transition by inducing store-operated calcium entry in lung adenocarcinoma. Oncotarget. 2016;7(45).

9. Dunn KMB, Lee PK, Wilson CM, Iida J, Wasiluk KR, Hugger M, et al. Inhibition of hyaluronan synthases decreases matrix metalloproteinase-7 (MMP-7) expression and activity. Surgery. 2009;145(3):322-9.

10. Li P, Xiang T, Li H, Li Q, Yang B, Huang J, et al. Hyaluronan synthase 2 overexpression is correlated with the tumorigenesis and metastasis of human breast cancer. International journal of clinical and experimental pathology. 2015;8(10):12101.

11. Zhang Z, Tao D, Zhang P, Liu X, Zhang Y, Cheng J, et al. Hyaluronan synthase 2 expressed by cancer-associated fibroblasts promotes oral cancer invasion. Journal of Experimental & Clinical Cancer Research. 2016;35(1):181.

12. Bernert B, Porsch H, Heldin P. Hyaluronan synthase 2 (HAS2) promotes breast cancer cell invasion by suppression of tissue metalloproteinase inhibitor 1 (TIMP-1). Journal of Biological Chemistry. 2011;286(49):42349-59.

13. Pangon L, Van Kralingen C, Abas M, Daly RJ, Musgrove EA, Kohonen-Corish MR. The PDZ-binding motif of MCC is phosphorylated at position− 1 and controls lamellipodia formation in colon epithelial cells. Biochimica et Biophysica Acta (BBA)-Molecular Cell Research. 2012;1823(6):1058-67.

14. Arnaud C, Sebbagh M, Nola S, Audebert S, Bidaut G, Hermant A, et al. MCC, a new interacting protein for Scrib, is required for cell migration in epithelial cells. FEBS letters. 2009;583(14):2326-32.

15. GUZIŃSKA-USTYMOWICZ K, Pryczynicz A, Kemona A. PTP4A3 expression increases strongly in lymph node metastases from colorectal carcinoma. Anticancer research. 2009;29(10):3913-6.

16. Pryczynicz A, Guziñska-Ustymowicz K, Chang X-J, Kisluk J, Kemona A. PTP4A3 (PRL-3) expression correlate with lymphatic metastases in gastric cancer. Folia Histochem Cytobiol. 2010;48(4):632-6.

17. Padua D, Massagué J. Roles of TGFβ in metastasis. Cell research. 2009;19(1):89-102.

18. Nadauld LD, Garcia S, Natsoulis G, Bell JM, Miotke L, Hopmans ES, et al. Metastatic tumor evolution and organoid modeling implicate TGFBR2 as a cancer driver in diffuse gastric cancer. Genome biology. 2014;15(8):1.

19. Wei C-Y, Tan Q-X, Zhu X, Qin Q-H, Zhu F-B, Mo Q-G, et al. Expression of CDKN1A/p21 and TGFBR2 in breast cancer and their prognostic significance. International journal of clinical and experimental pathology. 2015;8(11):14619.
