# Supplementary material for: Decomposing the subclonal structure of tumors with two-way mixture models on copy number aberrations
Source: PLoS One. 2018 Dec 12;13(12):e0206579. doi: 10.1371/journal.pone.0206579 (PMC6291075; doi:10.1371/journal.pone.0206579)
Supplement: S2 Table — (PDF) [file pone.0206579.s002.pdf]

Supplementing information

S2. Table List of reference genes in 11q13.3

Gene Symbol	Reference
CCND1	[1-3]
CTTN	[1-3]
DHCR7	[4]
FADD	[5-7]
FGF4	[8-10]
MYEOV	[8]
ORAOV1	[11, 12]
PPFIA1	[13]
SHANK2	[4]
TPCN2	[14, 15]

1. Ying J, Shan L, Li J, Zhong L, Xue L, Zhao H, et al. Genome-wide screening for genetic alterations in esophageal cancer by aCGH identifies 11q13 amplification oncogenes associated with nodal metastasis. PloS one. 2012;7(6):e39797.

2. Luo M-L, Wang M-R. CTTN (EMS1): an oncogene contributing to the metastasis of esophageal squamous cell carcinoma. Cell research. 2007;17(4):298-300.

3. Luo M-L, Shen X-M, Zhang Y, Wei F, Xu X, Cai Y, et al. Amplification and overexpression of CTTN (EMS1) contribute to the metastasis of esophageal squamous cell carcinoma by promoting cell migration and anoikis resistance. Cancer research. 2006;66(24):11690-9.

4. Hao J-J, Shi Z-Z, Zhao Z-X, Zhang Y, Gong T, Li C-X, et al. Characterization of genetic rearrangements in esophageal squamous carcinoma cell lines by a combination of M-FISH and array-CGH: further confirmation of some split genomic regions in primary tumors. BMC cancer. 2012;12(1):1.

5. Dent P. FADD the bad in head and neck cancer. Cancer biology & therapy. 2013;14(9):780-1.

6. Pattje W, Melchers L, Slagter-Menkema L, Mastik M, Schrijvers M, Gibcus J, et al. FADD expression is associated with regional and distant metastasis in squamous cell carcinoma of the head and neck. Histopathology. 2013;63(2):263-70.

7. Callegari CC, Cavalli IJ, Lima RS, Jucoski TS, Torresan C, Urban CA, et al. Copy number and expression analysis of FOSL1, GSTP1, NTSR1, FADD and CCND1 genes in primary breast tumors with axillary lymph node metastasis. Cancer Genetics. 2016.

8. Sugahara K, Michikawa Y, Ishikawa K, Shoji Y, Iwakawa M, Shibahara T, et al. Combination effects of distinct cores in 11q13 amplification region on cervical lymph node metastasis of oral squamous cell carcinoma. International journal of oncology. 2011;39(4):761.

9. Muller D, Millon R, Lidereau R, Engelmann A, Bronner G, Flesch H, et al. Frequent amplification of 11q13 DNA markers is associated with lymph node involvement in human head and neck squamous cell carcinomas. European Journal of Cancer Part B: Oral Oncology. 1994;30(2):113-20.

10. Qi L, Song W, Li L, Cao L, Yu Y, Song C, et al. FGF4 induces epithelial-mesenchymal transition by inducing store-operated calcium entry in lung adenocarcinoma. Oncotarget. 2016;7(45).

11. Li M, Cui X, Shen Y, Dong H, Liang W, Chen Y, et al. ORAOV1 overexpression in esophageal squamous cell carcinoma and esophageal dysplasia: a possible biomarker of progression and poor prognosis in esophageal carcinoma. Human pathology. 2015;46(5):707-15.

12. Kang JU, Koo SH. ORAOV1 is a probable target within the 11q13. 3 amplicon in lymph node metastases from gastric adenocarcinoma. International journal of molecular medicine. 2012;29(1):81.

13. Choi EJ, Yun JA, Jabeen S, Jeon EK, Won HS, Ko YH, et al. Prognostic significance of TMEM16A, PPFIA1, and FADD expression in invasive ductal carcinoma of the breast. World journal of surgical oncology. 2014;12(1):1.

14. Song Y, Li L, Ou Y, Gao Z, Li E, Li X, et al. Identification of genomic alterations in oesophageal squamous cell cancer. Nature. 2014;509(7498):91-5.

15. Salahshourifar I, Vincent-Chong VK, Kallarakkal TG, Zain RB. Genomic DNA copy number alterations from precursor oral lesions to oral squamous cell carcinoma. Oral oncology. 2014;50(5):404-12.
