# Supplementary figures and images for: Decomposing the subclonal structure of tumors with two-way mixture models on copy number aberrations
Source: PLoS One. 2018 Dec 12;13(12):e0206579. doi: 10.1371/journal.pone.0206579 (PMC6291075; doi:10.1371/journal.pone.0206579)

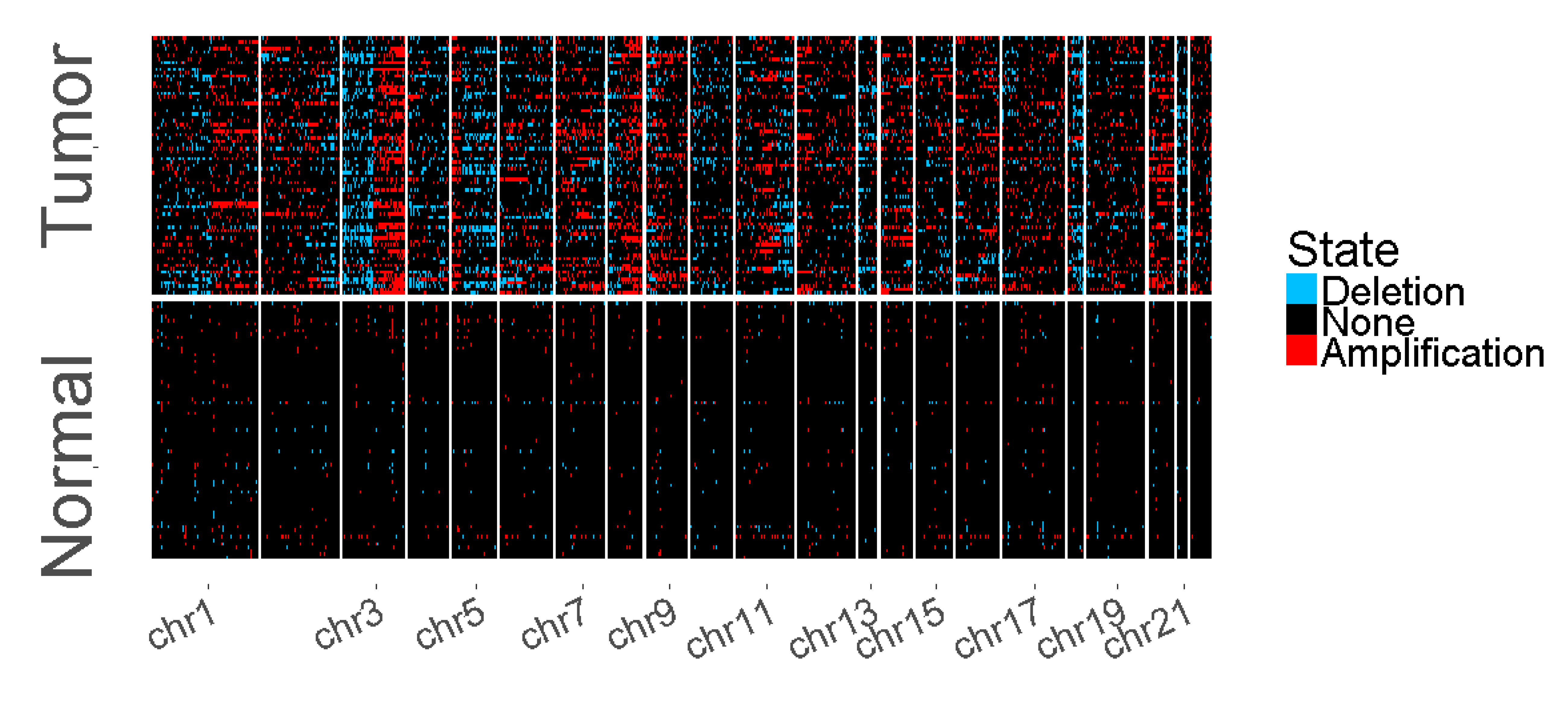

Supplement: S1 Fig — The estimated copy number states for the exons across the genome are presented in different colors. Light blue and red represent the deletion and amplification events, respectively. Black indicates no copy number changes. (TIFF) [file pone.0206579.s003.tiff]

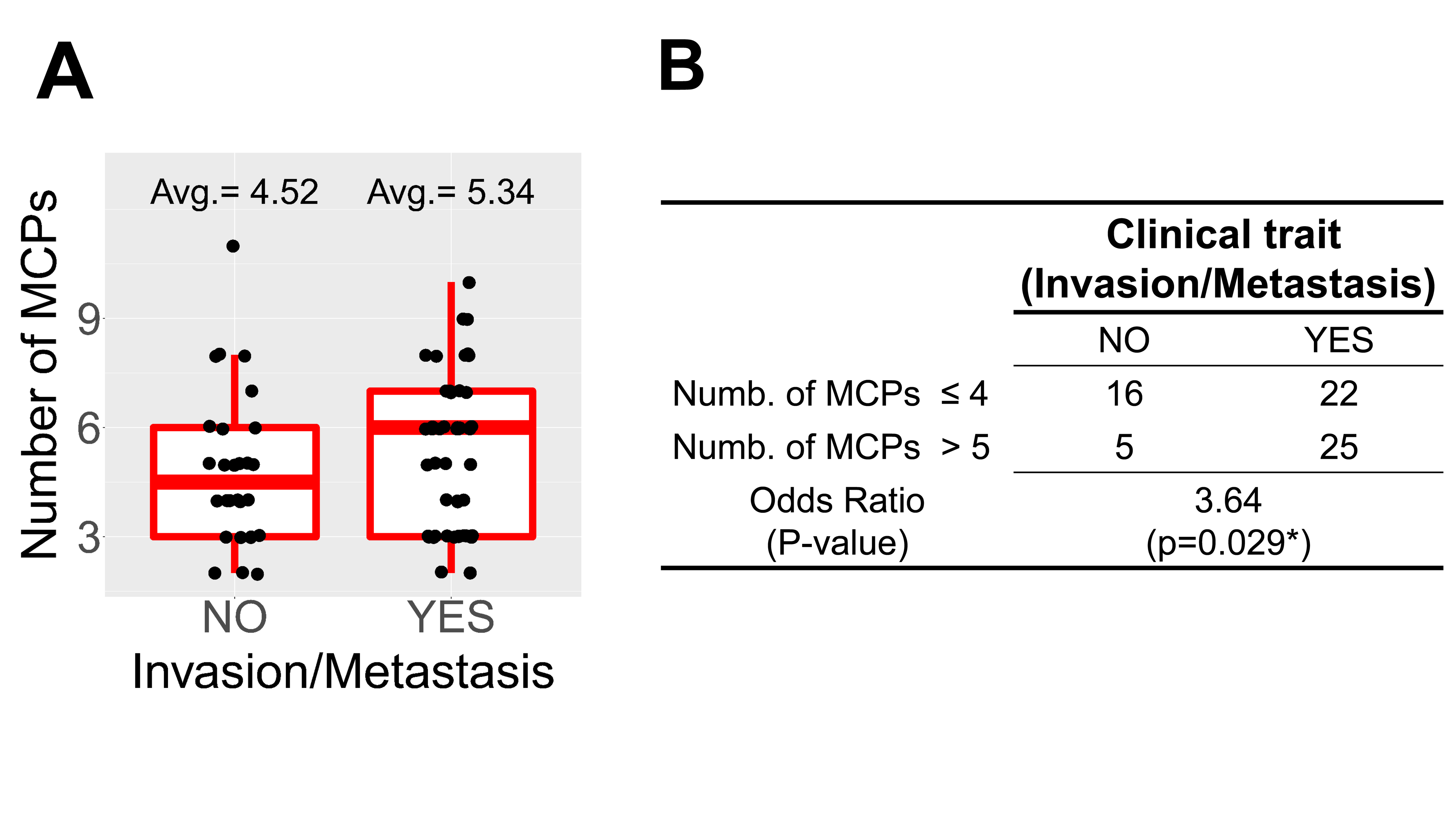

Supplement: S2 Fig — (A) The box plot for the number of MCPs with and without invasion or metastasis. The number of MCPs in each sample is represented by a black point jittered around the box. (B) Contingency table for dichotomization of tumor heterogeneity and clinical outcomes. (TIF) [file pone.0206579.s004.tif]

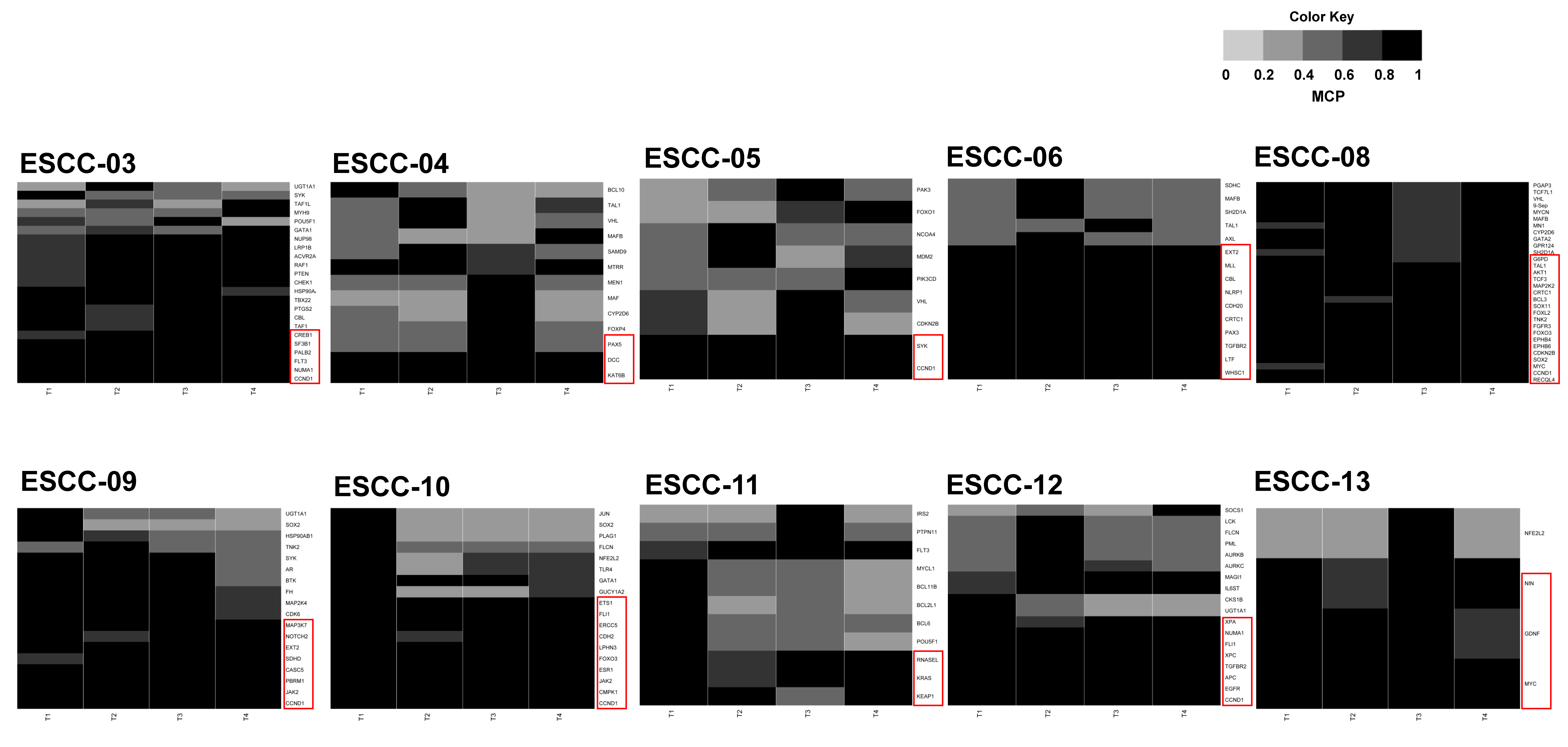

Supplement: S3 Fig — There are six MCP matrices. The color of each cell represents the MCP quantity of a gene for a given sample. The labels of rows indicate the gene symbols, and the labels of columns are region index A gene within the red rectangle is identified as the gene located on the trunk of an evolutionary tree. (TIFF) [file pone.0206579.s005.tiff]

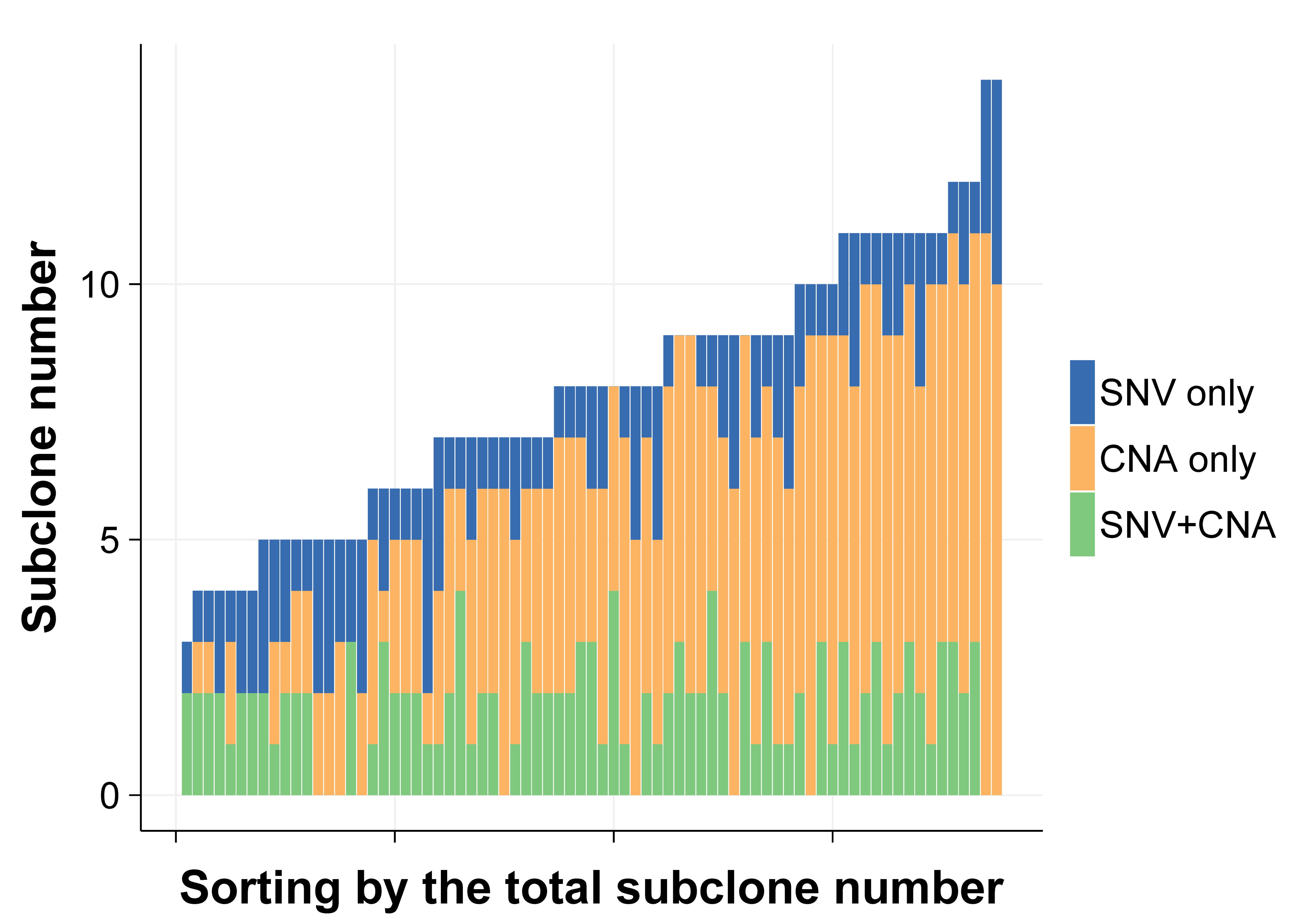

Supplement: S4 Fig — This figure summarizes the number of subclone we identified across 75 tumors by bar plot. The blue bar represents the subclones consisting of only SNVs, and the yellow one represents the subclones consisting of only CNAs. The green bar records the number of subclones consisting of both types of mutations. (TIF) [file pone.0206579.s006.tif]
