# Supplementary material for: Decomposing the subclonal structure of tumors with two-way mixture models on copy number aberrations
Source: PLoS One. 2018 Dec 12;13(12):e0206579. doi: 10.1371/journal.pone.0206579 (PMC6291075; doi:10.1371/journal.pone.0206579)
Supplement: S1 File — (DOCX) [file pone.0206579.s007.docx]

**Supplementary**

**Extended version to include SNV analysis**

CloneDeMix aims to explore tumor heterogeneity (TH) by inferring copy number aberration. The utility of CloneDeMix is not only for evaluating clonal structure caused by copy number change but also for providing a more precise copy number adjustment in SNV studies. Moreover, the model setting of CloneDeMix can be easily modified as a new version for inferring SNV in tumors. To complete the exploration of tumor heterogeneity, we further developed an extended approach to infer SNV by integrating the estimated copy number and the subclonal structure obtained from CloneDeMix. The flowchart of extended framework is depicted in Figure 1-1.This extended approach directly models variant allele count and normal allele count to infer cellular proportion by clustering all SNVs with the adjusted copy numbers. Following the concept of CloneDeMix modeling, we assume the allele count of each SNVs also follows a Poisson distribution, and its mean is proportional to the combination of the read count from different genetic mutation and the corresponding mutational cellular prevalence. The complete mean structure of Poisson model will be discussed in the next paragraph. Notably, the aforementioned genetic mutations include single nucleotide substitution and the copy number change.

As many studies have shown, the time order between SNV event and CNA event is essential for the statistical modeling in tumor heterogeneity studies. Therefore, to determine the mean structure of SNVs, pre-estimating the order of SNV event and CNA event is needed. All possible combinations of the mean structures resulted from different orders are listed in Table 1-1, and some examples are illustrated in Figure 1-2. The notations appeared in Table 1-1 are defined as follows. First, we assume $A_{l}$ and $B_{l}$ to be the normal allele count and variant allele count at locus-*l* respectively. In order to distinguish the use of MCP for copy number change in manuscript, we further use a mutational cellular prevalence for SNV to represent the fraction of the cells acquiring this SNV, and it is denoted as $p$. If an SNV is on a CNA region, we should take into account the corresponding MCP of CNA in the mean structure. We use $\hat{r}$ to represent the estimate of MCP for copy number change before we include the SNV analysis, and the copy number associated with the current SNV is denoted as $\hat{C}$. Now, we can model the normal allele count $A_{l}$ and the variant allele count $B_{l}$ as

$A_{l}\sim Poisson(\lambda_{A_{l}}(p))$,

$B_{l}\sim Poisson\left( \lambda_{B_{l}}(p) \right)$,

where $\lambda_{A_{l}}(p)$ and $\lambda_{B_{l}}(p)$ belong to one of the mean structures in Table 1-1. For instance, if locus-*l* satisfies the scenario of case 2-1, the mean structures will be

$\lambda_{A_{l}}(p)=\hat{a}_{l}\times\left[ \left( 2-p \right)+\left( \hat{C}-2 \right)\times\hat{r} \right]$ and

$\lambda_{B_{l}}(p)=\hat{a}_{l}\times p$.

where $\hat{C}$ and $\hat{r}$ are the estimated copy number and MCP obtained from CloneDeMix for pure CNA analysis, and $\hat{a}_{l}$ is a plug-in estimate of baseline from the paired normal tissue.

To determine which structure can better fit the data from locus-*l*, we introduced a new procedure as follows. First, we define a new notation $\Lambda$ to represent the set of mean structures and assume the pair ($\lambda_{A_{l}}^{*}$, $\lambda_{B_{l}}^{*}$) to be the optimal structure among $\Lambda$ for locus-*l*. We next use the product of two density functions ($Poisson\left( \lambda_{A_{l}}\left( p \right) \right)$ and $Poisson\left( \lambda_{B_{l}}\left( p \right) \right)$) as the objective function for optimization, and ($\lambda_{A_{l}}^{*}$, $\lambda_{B_{l}}^{*}$) is calculated by

($\lambda_{A_{l}}^{*}$, $\lambda_{B_{l}}^{*}$) = ${argmax}_{\left( \lambda_{A_{l}}, \lambda_{B_{l}} \right)\in\Lambda}Poisson\left( \lambda_{A_{l}}\left( p \right) \right)\times Poisson\left( \lambda_{B_{l}}\left( p \right) \right)$.

However, the objective function varies with p. To address this problem, we assume p to be uniformly distributed between 0 and 1 and integrate the above function with respect to p. Hence, the optimization function is rewritten as

($\lambda_{A_{l}}^{*}$, $\lambda_{B_{l}}^{*}$) = ${argmax}_{\left( \lambda_{A_{l}}, \lambda_{B_{l}} \right)\in\Lambda}\int_{0}^{1} Poisson\left( \lambda_{A_{l}}\left( p \right) \right)\times Poisson\left( \lambda_{B_{l}}\left( p \right) \right) dp.$

Consequently, the optimal mean structure of each locus can be selected by this optimization function.

Once we determine the mean structure of a locus, the final step in the extended framework is the estimation of *p*. We apply the one-way mixture clustering approach to infer *p* and use the EM algorithm to solve this problem. Because SNV and CNA can share the same subclone, the MCPs pre-estimated from the CNAs are fixed in the iteration procedure of the extended framework. The likelihood is formulated as

$\prod_{l} [ \sum_{k=1}^{K} \pi_{k}Poi\left( A_{l}|\lambda_{A_{l}}\left( \hat{r}_{k} \right) \right)\times Poi\left( B_{l}|\lambda_{B_{l}}\left( \hat{r}_{k} \right) \right)+$

$\sum_{s=1}^{S} \pi_{s}Poi\left( A_{l}|\lambda_{A_{l}}\left( p_{s} \right) \right)\times Poi\left( B_{l}|\lambda_{B_{l}}\left( p_{s} \right) \right) ]$,

where $\pi_{k}$ and $\pi_{s}$ are the weights of each cluster and $\sum_{k} \pi_{k}+\sum_{s} \pi_{s}=1$. The final number of clusters is also determined by AIC.

**Table 1-1. The mean structure of different cases**

| **Case 1. SNV only**  $A_{l}\propto2\times\left( 1-p \right)+1\times p=2-p$  $B_{l}\propto0\times\left( 1-p \right)+1\times p=p$ |
| --- |
| **Case 2-1. CNA (amplification) -> SNV (normal chromosome)**  $A_{l}\propto2\times\left( 1-\hat{r} \right)+\hat{C}\times\left( \hat{r}-p \right)+\left( \hat{C}-1 \right)\times p=\left( 2-p \right)+\left( \hat{C}-2 \right)\times\hat{r}$  $B_{l}\propto0\times\left( 1-\hat{r} \right)+0\times\left( \hat{r}-p \right)+1\times p=p$ |
| **Case 2-2. CNA (amplification) -> SNV (CNA chromosome)**  $A_{l}\propto2\times\left( 1-\hat{r} \right)+\hat{C}\times\left( \hat{r}-p \right)+\left( \hat{C}-1 \right)\times p=\left( 2-p \right)+\left( \hat{C}-2 \right)\times\hat{r}$  $B_{l}\propto0\times\left( 1-\hat{r} \right)+0\times\left( \hat{r}-p \right)+1\times p=p$ |
| **Case 2-3. CNA (deletion of one copy) -> SNV (normal chromosome)**  $A_{l}\propto2\times\left( 1-\hat{r} \right)+1\times\left( \hat{r}-p \right)+\left( 1-1 \right)\times p=\left( 2-p \right)-\hat{r}$  $B_{l}\propto0\times\left( 1-\hat{r} \right)+0\times\left( \hat{r}-p \right)+1\times p=p$ |
| **Case 3-1. SNV -> CNA (amplification of the variant allele)**  $A_{l}\propto2\times\left( 1-p \right)+1\times\left( p-\hat{r} \right)+1\times\hat{r}=\left( 2-p \right)$  $B_{l}\propto0\times\left( 1-p \right)+1\times\left( p-\hat{r} \right)+\left( \hat{C}-1 \right)\times\hat{r}=p+\left( \hat{C}-2 \right)\times\hat{r}$ |
| **Case 3-2. SNV -> CNA (amplification of the normal allele)**  $A_{l}\propto2\times\left( 1-p \right)+1\times\left( p-\hat{r} \right)+\left( \hat{C}-1 \right)\times\hat{r}=\left( 2-p \right)+\left( \hat{C}-2 \right)\times\hat{r}$  $B_{l}\propto0\times\left( 1-p \right)+1\times\left( p-\hat{r} \right)+1\times\hat{r}=p$ |
| **Case 3-3. SNV -> CNA (deletion of one copy of the normal allele)**  $A_{l}\propto2\times\left( 1-p \right)+1\times\left( p-\hat{r} \right)+\left( 1-1 \right)\times\hat{r}=\left( 2-p \right)-\hat{r}$  $B_{l}\propto0\times\left( 1-p \right)+1\times\left( p-\hat{r} \right)+1\times\hat{r}=p$ |
| **Case 3-4. SNV -> CNA (deletion of one copy of the variant allele)**  $A_{l}\propto2\times\left( 1-p \right)+1\times\left( p-\hat{r} \right)+1\times\hat{r}=2-p$  $B_{l}\propto0\times\left( 1-p \right)+1\times\left( p-\hat{r} \right)+0\times\hat{r}=p-\hat{r}$ |
| **Case 3-5. SNV -> CNA (deletion of two copies)**  $A_{l}\propto2\times\left( 1-p \right)+1\times\left( p-\hat{r} \right)+0\times\hat{r}=\left( 2-p \right)-\hat{r}$  $B_{l}\propto0\times\left( 1-p \right)+1\times\left( p-\hat{r} \right)+0\times\hat{r}=p-\hat{r}$ |
| **Case 4-1. SNV and CNA are disjoint (amplification)**  $A_{l}\propto C\times\hat{r}+2\times\left( 1-p-\hat{r} \right)+1\times p=\left( 2-p \right)+\left( \hat{C}-2 \right)\times\hat{r}$  $B_{l}\propto0\times\hat{r}+0\times\left( 1-p-\hat{r} \right)+1\times p=p$ |
| **Case 4-2. SNV and CNA are disjoint (deletion of one copy)**  $A_{l}\propto1\times\hat{r}+2\times\left( 1-p-\hat{r} \right)+1\times p=\left( 2-p \right)-\hat{r}$  $B_{l}\propto0\times\hat{r}+0\times\left( 1-p-\hat{r} \right)+1\times p=p$ |
| **Case 4-3. SNV and CNA are disjoint (deletion of two copies)**  $A_{l}\propto0\times\hat{r}+2\times\left( 1-p-\hat{r} \right)+1\times p=\left( 2-p \right)-2\times\hat{r}$  $B_{l}\propto0\times\hat{r}+0\times\left( 1-p-\hat{r} \right)+1\times p=p$ |


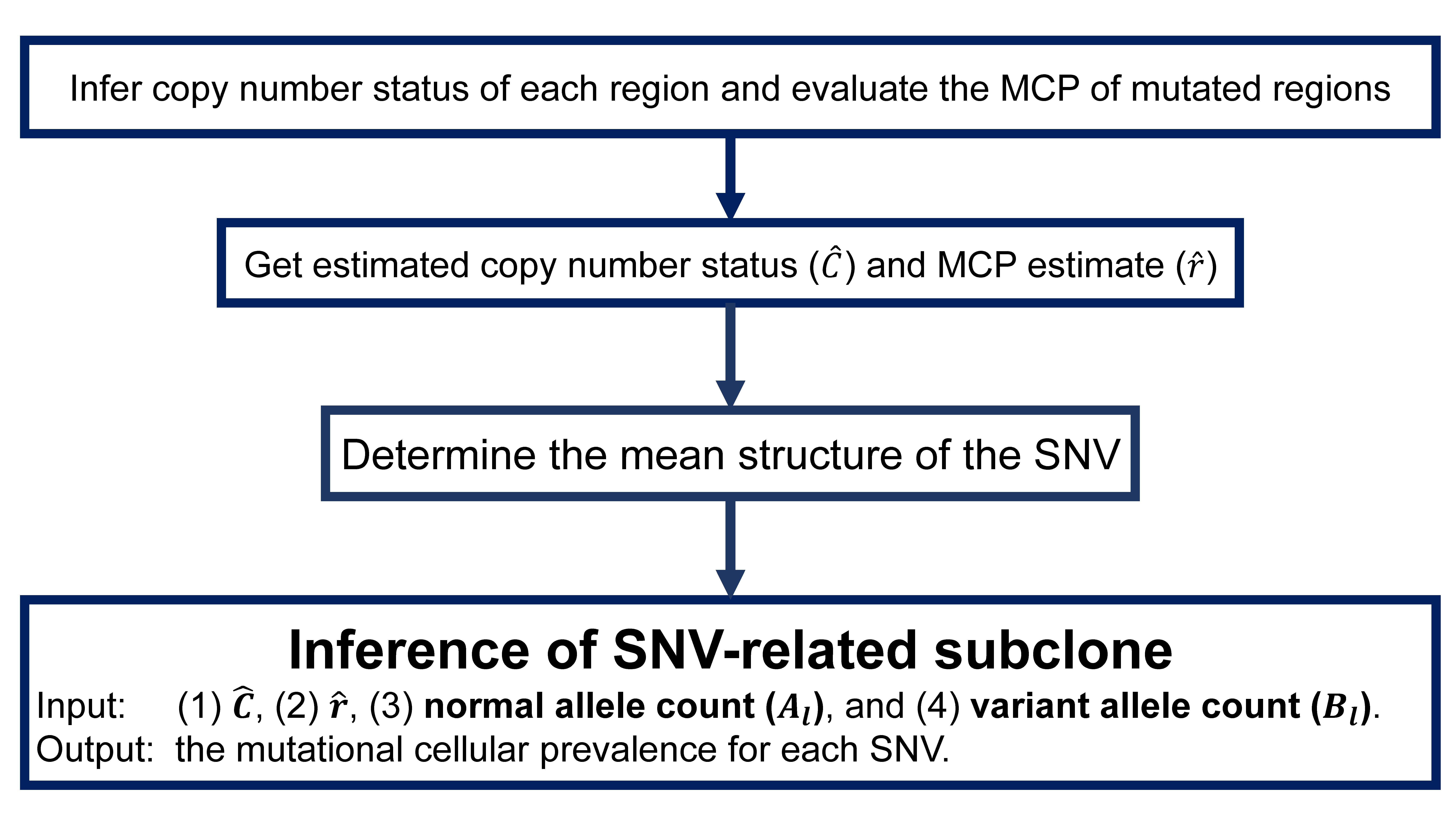


**Figure 1-1. Flowchart of the extended framework**


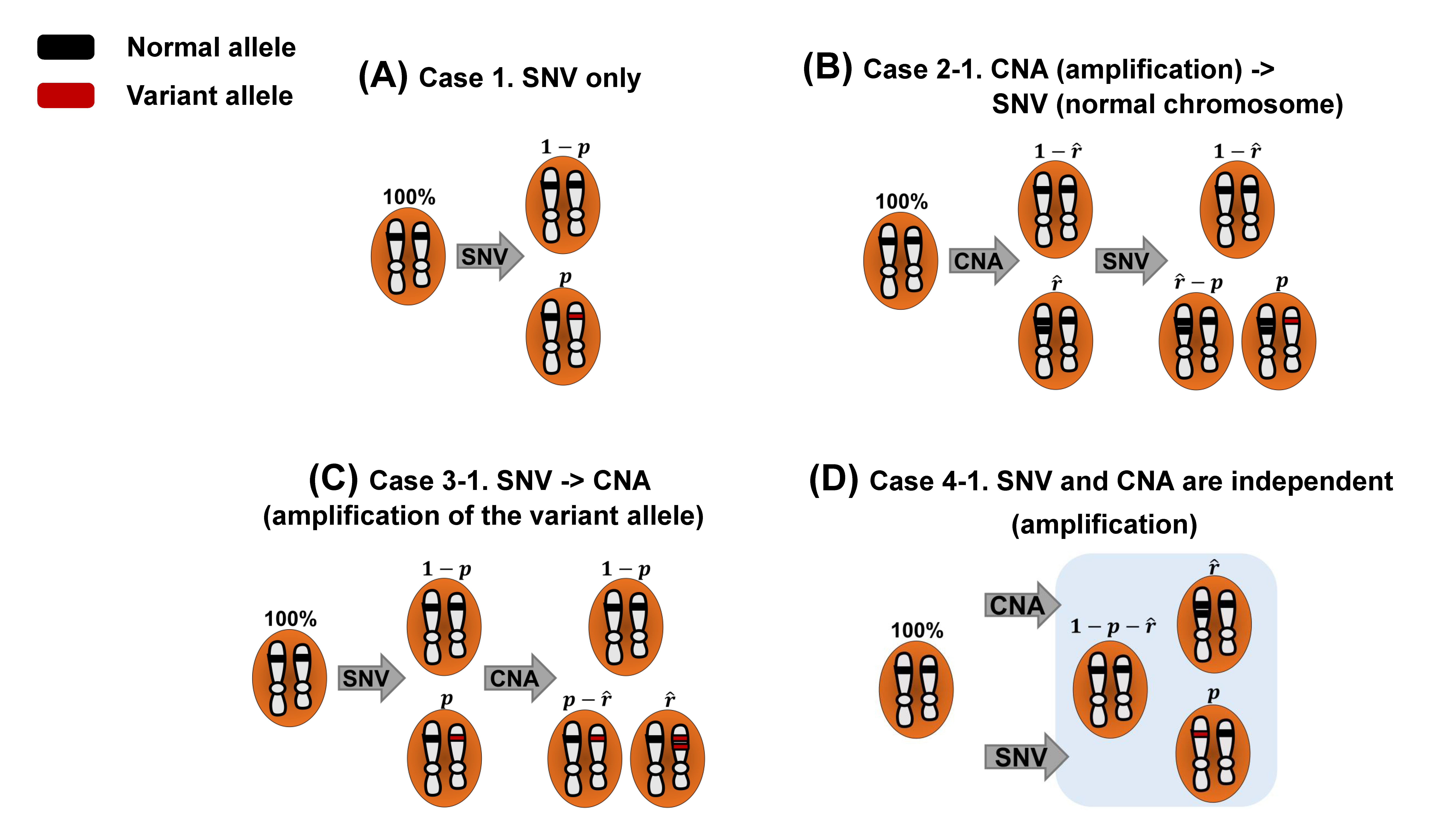


**Figure 1-2. Illustrations of the relation between SNV and CNA.**

There are four examples to demonstrate different evolutionary order for SNV analysis in a tumor. These examples only consider copy number amplification. (A) A subclone acquired SNV variants only and proliferated to proportion *p*. (B) A subclone first acquired a CNA to the proportion $\hat{r}$ and then split into another subclone acquiring additional SNV. (C) A subclone first acquired an SNV to the proportion *p* and then split into another subclone acquiring an additional CNA. (D) The tumor independently developed two subclones. One acquired an SNV and the other acquired a CNA. Both are located at the same chromosomal position.
